# Parameter estimation and inference for stochastic reaction-diffusion systems: application to morphogenesis in D. melanogaster

**DOI:** 10.1186/1752-0509-4-21

**Published:** 2010-03-10

**Authors:** Michael A Dewar, Visakan Kadirkamanathan, Manfred Opper, Guido Sanguinetti

**Affiliations:** 1 Department of Applied Physics and Applied Mathematics, Columbia University, New York, USA; 2 Department of Automatic Control and Systems Engineering, University of Sheffield, Sheffield, UK; 3 Fakultät Elektrotechnik und Informatik, Technische Universität Berlin, Berlin, Germany; 4 Department of Computer Science, University of Sheffield, Sheffield, UK; 5 ChELSI Institute, Department of Chemical and Process Engineering, University of Sheffield, Sheffield, UK; 6 School of Informatics, The University of Edinburgh, Edinburgh, UK

## Abstract

**Background:**

Reaction-diffusion systems are frequently used in systems biology to model developmental and signalling processes. In many applications, count numbers of the diffusing molecular species are very low, leading to the need to explicitly model the inherent variability using stochastic methods. Despite their importance and frequent use, parameter estimation for both deterministic and stochastic reaction-diffusion systems is still a challenging problem.

**Results:**

We present a Bayesian inference approach to solve both the parameter and state estimation problem for stochastic reaction-diffusion systems. This allows a determination of the full posterior distribution of the parameters (expected values and uncertainty). We benchmark the method by illustrating it on a simple synthetic experiment. We then test the method on real data about the diffusion of the morphogen *Bicoid *in *Drosophila melanogaster*. The results show how the precision with which parameters can be inferred varies dramatically, indicating that the ability to infer full posterior distributions on the parameters can have important experimental design consequences.

**Conclusions:**

The results obtained demonstrate the feasibility and potential advantages of applying a Bayesian approach to parameter estimation in stochastic reaction-diffusion systems. In particular, the ability to estimate credibility intervals associated with parameter estimates can be precious for experimental design. Further work, however, will be needed to ensure the method can scale up to larger problems.

## Background

Reaction-diffusion systems play a fundamental role in modelling spatio-temporal dynamics in systems biology. Originally introduced by Turing [1] over 50 years ago to provide a microscopic explanation of morphogenesis, they have been extensively used to explain pattern and organ formation in animals and plants [2,3], as well as other spatio-temporal processes such as quorum sensing in bacterial biofilms [4]. The deterministic reaction-diffusion system is given by a system of partial-differential equations(1)

where Δ represents the Laplacian operator (second derivative in the spatial directions). Here, **c **is a vector of concentrations of chemical species, *D *is a diagonal matrix of diffusion coefficients and *f *encodes the reaction terms between different species.

An example of a systems biology application of this type of models is the formation of morphogen gradients during development. In the simplest case, **c **represents the concentration of the morphogen across space, which can diffuse through the embryo over time and decays at a rate independent of its position. If we assume that production of **c **can happen only in a specific region of the embryo, then, after a transient period, the steady state solution will exhibit a gradient in the concentration of **c**. While this is a very simple example, it is already non-trivial due to the interplay of spatial and temporal dynamics. This example also highlights another important feature of the reaction-diffusion systems encountered in systems biology, *i.e*. the fact that they necessarily will involve low counts of molecules. An embryo in the early stages of development may consist of only a handful of cells; even if maternal deposits of the morphogen consist of thousands of proteins, the counts of morphogen proteins in cells far from the deposit will necessarily start very low. At these count numbers, stochastic fluctuations may become important, and it has been argued that stochastic reaction-diffusion models are best suited to describe biological spatio-temporal systems [5].

By far the most used tool when dealing with stochastic processes is simulation. Gillespie's algorithm [6] provides an elegant and efficient tool to simulate chemical reactions with *K *species of interacting individuals. Its basic ideas can be extended to spatio-temporal systems by discretising space into a number of *N *bins and then simulating the system's behaviour as a chemical reaction with *N *× *K *species, where diffusion in continuous space is replaced with a discrete interaction between neighbouring bins (for a review, see *e.g*. [7]). This procedure is partly motivated by its computational simplicity, but also by the fact that data about the precise location of a particle is very rare, while an approximate count of particles within a certain region is much easier to obtain. While simulation is certainly a powerful tool to get insights on the plausible dynamics of the system, estimation of the systems parameters is often difficult. While in deterministic systems optimisation based approaches have been shown to yield some success [8,9], the problem in stochastic systems is compounded by the fact that the true state of the system is also a random variable, and its distribution must be inferred (the so-called *state inference *problem). Parameters are often fitted using heuristics (*e.g*. by comparison with steady states [5]) which do not have any guarantee of capturing the correct dynamical behaviour of the system.

In this paper we present an approximate solution of both the state inference and the parameter estimation problems for stochastic reaction-diffusion systems. We exploit the idea of discretising space and model the spatio-temporal process as a finite number of reaction systems happening in spatial bins which can communicate with each other. We draw upon a recently proposed framework for approximate inference in Markovian stochastic jump processes [10] to tackle the inference problem in discrete-space, continuous time reaction diffusion systems. The Bayesian nature of our approach means that we can provide full probability distributions over the inferred parameters and states, not just point estimates. We initially evaluate our approach on a simple but realistic synthetic dataset, to assess the accuracy and identifiability of our system. As previously reported for deterministic systems [9], we find that some global identifiability issues exist, but nevertheless the results can yield valuable information. We then investigate the case study of *Bicoid *gradient formation in *Drosophila melanogaster *[5]. The inferred parameters are reasonable; interestingly, the precision with which the parameters can be inferred varies dramatically between the different parameters. This gives a useful way of ranking possible parameters in terms of information content, suggesting that experimental determination of highly uncertain parameters should be prioritised. The rest of the paper is organised as follows: in the next section, we present our model of *Bicoid *dynamics, articulate the scientific question we are trying to answer, and present results of our approach both on a simulated and real developmental data set. In the conclusion, we continue the discussion of our results, emphasizing the novelty with respect to existing approaches. We then present in the methods section the detailed derivation of our inference algorithm.

## Results and Discussion

### Basic Model of Bicoid dynamics

We consider the stochastic version of the reaction-diffusion system described in equation(1). In the case of *Bicoid*, we only consider a single molecular species diffusing and reacting through the embryo. We further exploit the axial symmetry of the embryo and consider a single spatial dimension. The stochastic model can therefore be thought of as a many-body system where particles can diffuse in space at a constant rate. *Bicoid *proteins can be produced in the anterior region of the embryo as mRNA deposited by the mother is translated, and proteins can decay anywhere in the embryo with constant rate.

A common way to model these spatio-temporal systems is to use a compartmentalised approach: space is divided into a number *N *of identical bins which are spatially homogeneous and can only communicate with neighbouring bins. Denoting with *x*_*i *_the number of *Bicoid *particles, the system can be described by a set of chemical reactions(2)

The first equation represents diffusion between neighbouring bins. This happens with a rate  where *D *is the diffusion constant and *h *is the width of the bins making up the system. Notice that this reaction is reversible, *i.e*. diffusion can happen in both directions. The second equation represents production of *Bicoid *proteins at a rate *k*_2_; in our model this happens only in the first bin (anterior region) where the maternal mRNA deposit is localised. Finally, the third reaction represents protein decay. All of the parameters have the same dimensions of inverse times.

Mathematically, the stochastic dynamics of chemical reactions at very low concentrations is conveniently described using the formalism of Markov Jump Processes (MJP). Exact sampling from MJPs is easily achieved using Gillespie's algorithm [6]. Given parameters and an initial state, this allows us to simulate the behaviour of the system over a period of time. Here, however, we are interested in the inverse problem: we observe the system at a discrete set of time points, obtaining noisy counts of the numbers of proteins in each bin. From these, we would like to infer the true continuous time trajectory of the system (state inference problem) and estimate the parameters of the model.

Exact statistical inference for MJPs is known to be computationally very intensive [11], ruling out even small-sized systems. Our approach will use a variational approximation to the inference problem which gives a reasonable accuracy with very contained computational costs [10]. This approach allows us to obtain a full posterior distribution over both the process and the parameters. We will detail our mathematical approach in the methods section; we now present some results on simulated and real data. While the mathematical theory is formulated in the general case of *K *interacting species, we will only deal with the case *K *= 1 in the experiments due to its relevance to the *Bicoid *morphogenesis. We refer the reader to [10] for an example with *K >*1 (but with no spatial dimensions).

### Synthetic data

In order to validate our approach, we generated synthetic data from a stochastic reaction-diffusion process using a compartment-based Gillespie algorithm. The reactions system we used for simulation is given in 2, where we fixed the number of bins to be eight.

For this examples, the reaction rates for anterior production and decay are chosen to be *k*_2 _= 0.4, *k*_1 _= 0.0001; the diffusion parameter is set to *d *= 0.01. This set of parameters was found to give sample trajectories which were qualitatively similar to those observed in the real data. Gamma priors with shape coefficient 2 were chosen for all the parameters; these were judged to be vague enough not to bias excessively the results. As we often have experimental estimates at least of the order of magnitude of the parameters, we chose the scale parameter of the Gammas so that most of the prior probability mass was concentrated at the right order of magnitude of the parameters.

The process is simulated using Gillespie's algorithm over 2000 time points (the time units in the simulation). The system reaches an approximate steady state towards the end of the simulation. The algorithm is initialised with zero particles in each bin. Fifteen equally spaced noisy observation samples are then taken from the first 1500 time points, forming the data set to be used for inference. The posterior process is initialised as a constant process with mode at the mean value of the observations. Ten samples from the same reaction-diffusion process were used, and the parameters where initialised at random from uniform distributions centred on the true value and with width chosen to cover variations of plus/minus 50%.

**Figure 1:**
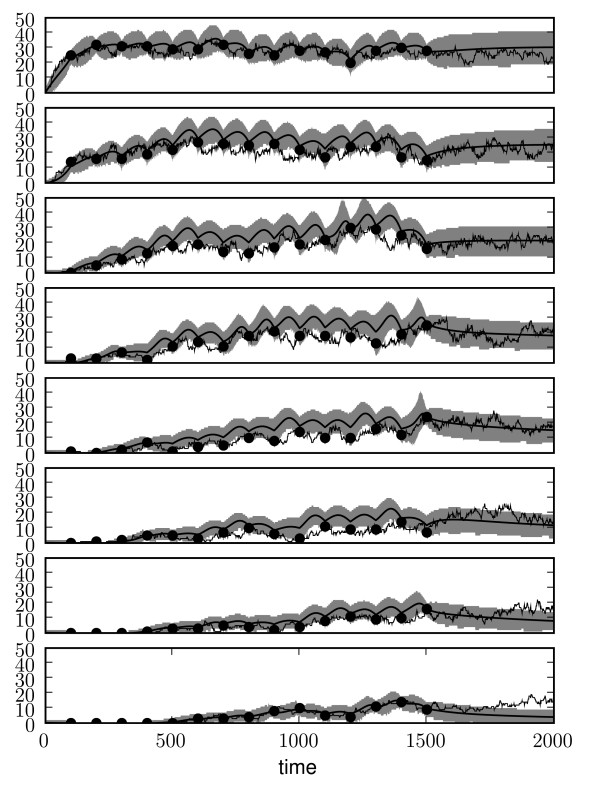
**State inference results on synthetic data**. Posterior synthetic spatio-temporal process at each of the eight spatial locations. The top to bottom plots correspond to left to right spatial locations, such that the leftmost bin, in which there is production, is shown at the top, and the rightmost bin is shown at the bottom. The thicker solid line represents the posterior mode, the grey area represents the 95% confidence interval and the thin line is the true path from which the data was sampled. The green crosses show the noisy observed data points. Notice that the posterior mode for the last bin is always on the ground state (no particles present). The *y *axis represent particle counts in the bin, the *x *axis time.

The results of the state inference for one of these runs are shown in Figure 1. Spatial bins are shown top to bottom, corresponding to left to right spatial locations. The top plot shows the leftmost spatial location, in which the particles are generated, and the bottom plot shows the rightmost spatial location. The thicker solid line shows the mean of the posterior process; the grey area the 95% confidence interval. The black points show the noisy observations and the thin line shows the true path from which the observations were taken. While the inferred posterior is in general in good agreement with the process, it seems to overestimate it in some bins. The fact that the prior process has very few parameters might explain this as the system is heavily constrained.

### Parameters estimation and identifiability

Parameter estimation in reaction-diffusion problems is known to suffer from identifiability issues even in the deterministic case [9]. The main difficulty is that both the production and decay terms are always coupled with the diffusion constant. This introduces correlations that are potentially very difficult to disentangle. Secondly, rescaling all the parameters by a common factor only has the effect of changing the time the system takes to reach steady state. Given the low particles counts we are considering, the stochastic fluctuations at steady state are of comparable magnitude to the average values. It is therefore unrealistic to expect to be able to obtain an accurate estimate of the time the system takes to reach steady state, which may lead in the parameter estimates being systematically scaled by a multiplicative constant. Finally, we should point out that the factorised approximation we make to compute the posterior process can sometimes lead to an underestimation of the true variability (see the Methods section for details). Therefore, the error bars estimated with our approach will in general be an underestimation of the true error bars.

**Figure 2:**
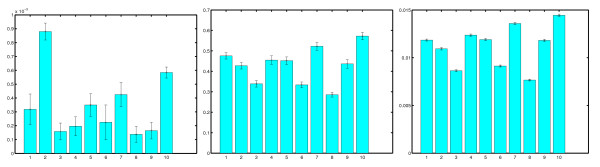
**Parameter estimation results on synthetic data**. Parameter estimation results for ten independent simulations of the same reaction-diffusion process with parameters *k*_2 _= 0.4, *k*_1 _= 0.0001 and *d *= 0.01. The three panels show the results for *k*_1 _(left), *k*_2 _(centre) and *d *(right). Notice the strong correlation between the parameters *k*_2 _and *d*, as well as the systematic underestimation of the error bars. Estimates of the parameter *k*_1 _(decay rate) are inaccurate, due to the difficulty in distinguishing the effects of decay from those of diffusion (see main text).

The results of the parameter estimation on the ten independent simulations are given in Figure 2. The left panel shows the results for *k*_1 _(decay rate, true value 0.0001), the middle panel the results for *k*_2 _(anterior production rate, true value 0.4) and the right panel the results for *d *(diffusion rate, true value 0.01). A number of things need to be noticed. First of all, estimates of *k*_1 _are largely inaccurate. This is not surprising, as the effects of decay are difficult to distinguish from the effects of diffusion in our model (both processes result in a particle leaving a bin). As the diffusion constant is two orders of magnitude greater than the decay constant, its effect will be largely negligible, rendering this parameter unidentifiable. Secondly, the results for *k*_2 _and *d *show a striking correlation; as mentioned before, simultaneous rescaling of production and decay will only result in a change in the time needed to achieve steady state, which is inherently difficult to estimate in stochastic processes. However, overall the approach returns a very reasonable estimate for both *k*_2 _and *d*. Finally, as mentioned before, the errobars associated with the estimates are generally underestimated; notice however that the error bars relative to *k*_2 _estimates are much bigger than the ones relative to *d *estimates, mirroring the fact that *k*_2 _is only active in one bin and hence harder to estimate.

### Real data

To test our model on real data we used *in situ *protein expression levels for the protein *Bicoid *at cleavage stage 14A in the *Drosophila *embryo. This system was the focus of a recent study [5] where the stochastic reaction-diffusion system was simulated using a compartment-based Gillespie algorithm with 100 bins. The parameters in this study were initialised by fitting to the steady state, taken to be given by the last time point. The data was obtained from the FlyEx database ([12], available from http://flyex.ams.sunysb.edu/flyex) and consists of six recordings of the *Bicoid *protein intensity during the diffusion of the morphogen, measured at 100 locations across the embryo. From this set, eight equally spaced locations were sampled forming a data set of eight spatial locations with six time points each. The time points are from equally spaced *time classes*, *i.e*. key times during the cleavage cycle identified by the curators of the data set through image analysis citePoustelnikova:database04. These can be thought of equally spaced in *developmental time*, although in general they are not equally spaced in real time. Therefore, the units of our parameters in this case will be the inverse of the time classes units. The choice to consider only a single cleavage cycle was dictated by the need to minimize the effects of growth and developmental changes on the system (which we do not explicitly model). The recorded intensities were reported in arbitrary units, therefore it is difficult to assign precise particle counts to these measurements. We chose to scale the data in order to give population levels between 0 and 60 particles at each location. This is motivated essentially by computational reasons (large particle counts slow down the algorithm). Although it does result in unrealistically low protein numbers (approximately 500 in the whole embryo), it can be justified assuming that what we model is the process in a small tube in the centre of the embryo. To model the noise introduced by this assumption, as well as the measurement noise, we assume that the observations are randomly distributed around the true value of the process with an exponentially decaying distribution (following closely [10]). The process was initialised using the first samples at each observation location, leaving five remaining points in each bin. We used the same model form as above, with initial parameter estimates of *k*_2 _= 0.05, *k*_1 _= 0.001 and *d *= 0.001. Again, the mode of the posterior is initialised at the mean value of the observations, and Gamma priors are placed over the parameters with a scale equal to the initial parameter estimates.

**Figure 3:**
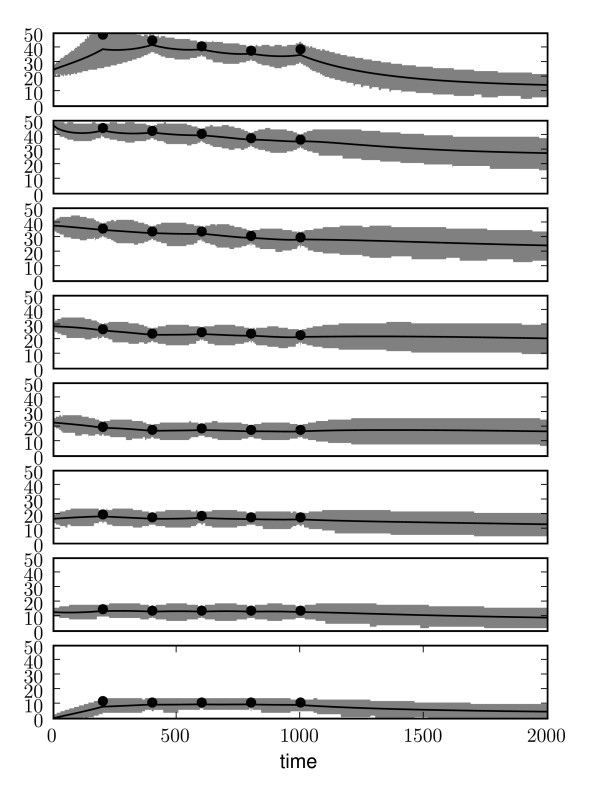
**State inference results on real data**. Posterior *Bicoid *reaction-diffusion process across eight spatial locations. The solid line represents the mean, the grey area represents the 95% confidence interval. The black points show the noisy observed data points. The *y *axis represent particle counts in the bin, the *x *axis time (in temporal class units, see main text).

The results are shown in Figure 3. The posterior process is shown to provide a good fit across the majority of data points, though the inferred model is unable to fully capture the fast dynamics associated with the *Bicoid *intensity in the first spatial location (the posterior mean is systematically lower than the observations). The very sharp rise in morphogen suggests spatial edge effects that are not captured by our model formulation. At steady state the predicted posterior process describes the expected morphogen gradient across the embryo, which enables the subsequent development of the *French-Flag *pattern. The inferred parameters for the model are *k*_1 _= 8 ± 4 × 10^-5^, *k*_2 _= 5 ± 2 × 10^-2 ^and *d *= 1.8 ± 0.1 × 10^-3^. As was observed previously, uncertainty over the parameter estimates can vary greatly. This can have useful repercussions when designing new experiments. For example, we see that the uncertainty over the diffusion parameter *d *is much smaller than the uncertainty over the other parameters. This makes sense since, for all the bins but the first, the rate of increase is determined solely by the diffusion constant and hence it can be accurately estimated from the data. The production and decay parameters, instead, have much broader distributions. Therefore, this would suggest that measuring the decay rate would significantly reduce our overall uncertainty, whilst measuring the diffusion constant would contribute very little extra knowledge about the dynamics of the system.

## Conclusions

Parameter estimation problems are becoming increasingly important in systems biology. While for deterministic systems methods based on optimisation have generally been yielding good results, there are no equivalent methods for stochastic systems, and widely used heuristics do not offer guarantees of accuracy. In this contribution, we present an approach to state inference and parameter estimation for stochastic reaction-diffusion systems. We focus on the important case study of *Bicoid *dynamics in *Drosophila melanogaster*. Our results show that inference in these systems is possible, even if parameter estimation suffers from some identifiability issues similar to those encountered in deterministic reaction-diffusion systems [9]. To our knowledge, this is the first time a Bayesian approach is proposed to perform inference in stochastic reaction-diffusion systems. Therefore, it is difficult to assess its quality in a comparative manner; the natural comparison would be with sampling based schemes such as [11], but their computational intensity rules out the application to systems of even moderate size like the one we consider. Stochastic reaction-diffusion models have been investigated in the context of *Bicoid *diffusion in a number of studies. For example, Wu *et al *[5] conducted a large scale simulation study of the process using Gillespie's algorithm for a compartmentalised system. Perhaps closer to our approach is the recent work of Lepzelter and Wang [13], who investigate the same biological problem by solving the reaction-diffusion master equation at steady state. While their approach leads to valuable insights in the nature of the intrinsic noise involved in the process, they do not address the issue of parameter estimation, and the steady state assumption limits its usefulness in describing dynamical processes.

While the results we reported are in our view encouraging, there are a number of improvements and generalisations which would be of interest. Firstly, efficient strategies are still required in order to tackle large scale systems; the simulation study in [5] employed 100 bins with average number of particles per bin in the low hundreds, which would be computationally very intensive using our approach. While coding economies could be made, alternative strategies based on quadratures could be useful. Another important extension would be to model several different proteins interacting, so that the reaction rates become non-linear functions of the state of the system. While handling non-linear systems is in principle not a problem for our approach, the increase in the number of species will again lead to substantially higher computational overheads.

## Methods

In this section we briefly review the mathematical foundations of Markov Jump Processes, as well as describing our approach to inference in these systems. We start by reviewing the stochastic theory of chemical reactions; we do this in the general case where many species of interacting particles are present, even if in our application only one species is considered. We then describe reaction-diffusion processes and how the compartmentalisation works. In particular, we should stress here that dividing space into *N *compartments is equivalent to replacing a single species existing in (inhomogeneous) space with *N *species living in a well-stirred mixture (each species being the population of a bin). Therefore, the variational approach described for chemical reactions with *K *species can be immediately transferred to a reaction-diffusion system involving one species and *K *spatial bins.

### Chemical reactions

We briefly review here the kinetic theory of chemical reactions and the underlying mathematical formalism of Markov jump processes. We assume that the system consists of a well-stirred mixture of *K *species *X*_1_, ..., *X*_*K *_of interacting particles, with *x*_*d*_(*t*) *d *= 1, ..., *K *being the number of particles of species *d *at time *t*. Assuming a Maxwell distribution for the velocities of the particles and assuming for simplicity that the stoichiometric coefficients are all 1, the probability of a reaction in which a particle of species *d*_1 _reacts with a particle of species *d*_2 _to form a particle of species *d*_3 _occurring in the infinitesimal time *δt *is given by [6]

where  is the kinetic rate of the reaction.

Markov jump processes (MJPs) provide a convenient mathematical formalism to model this type of processes. A MJP is a family of discrete random variables indexed by time; it is characterised by its transition rates *f *(**x'|x**) defined as(3)

Another important quantity is the marginal distribution *p*_*t *_(**x**) that the system is in a particular state at a certain time *t*. The relationship between the transition rates and the marginals is given by the *Master equation*(4)

This is the analogue of the forward Fokker-Planck equation of stochastic differential equations. It is worth remarking that in general the master equation is a huge system of linear ODEs with *S*^*K *^equations, where *S *is the maximum number of particles that can exist in any one species. Therefore, for all but the simplest reaction systems, direct solution of the master equation is not a viable option.

### Reaction-diffusion systems

The above description of chemical reactions relies on the central assumption that the reactants are in a well-stirred mixture, so that the spatial distribution of each species is uniform. However, in many applications, this is an unrealistic assumption and the spatial distribution of the particles has to be kept into account.

Stochastic reaction-diffusion systems are many-particle systems in which each individual particle performs diffusion in space, and simultaneously chemical reactions can happen when particles collide (or spontaneously in the case of decay and spontaneous production). In the isotropic case with no external drift, the diffusion of each particle is given by the Smoluchowski equation

where **z **is the position vector of the particle and *D *is the diffusion constant. Since the number of particles in the system is variable, the expression relating marginals and (diffusion and reaction) rates analogous to the Master or Fokker-Planck equation needs to be formulated in Fock space [14] and is generally much harder to handle. Furthermore, in many important applications the spatial resolution of the data is not accurate enough to allow the tracking of every single particle in the system.

For both these reasons, a common approach is to discretise space into *N *bins, and then treat the discretised system as a Markov jump process with as many species as the original number of species *K *times the number of bins. The transition rates for this process are given by the sum of the reaction transition rates plus a diffusion part representing the fact that numbers in a bin can change due to influx (departure) of particles from (to) the neighbouring bins. Explicitly, the transition rates *f *for the reaction-diffusion process are given by(5)

Here  represents the number of particles of species *m *in bin *i*, *f*_*R *_are the rates due to reactions going on in the *i*-th bin and  where *h *is the width of the bin (the factor 2 in the second equation is due to diffusion happening through both walls of the bin).

### Variational inference and parameter estimation

Opper and Sanguinetti [10] recently proposed a variational approach to approximately compute a posterior MJP given discrete observations with independent and identically distributed noise. The approach was based on approximating the posterior process with a factorised process where the effect of the interactions between species were replaced by a mean-field approximation. This was interleaved with parameter estimation steps in an Expectation-Maximization (EM) algorithm. This allowed the authors to reduce the complexity of the problem from exponential to linear in the number of species and to set up a forward-backward procedure to iteratively determine the rates and marginals of the approximating process. Let **y **denote the observed data, which are related to the true state of the system via a noise model *p *(**y***j***x**). The variational approximation relies on the minimisation of the *variational free energy*(6)

where

is the *Kullback-Leibler *divergence, an information theoretic measure of dissimilarity between distributions. Here ***θ ***denotes collectively the parameters of the model (decay, diffusion constant, *etc*.) plus any parameters contained in the observation noise model. We use the notation **x**_0:*T *_to denote the whole process as opposed to the random variables **x**(*t*) (whose distribution is given by the marginal). It can be shown that the bound in (6) is saturated if and only if the distribution *q *(the *approximating distribution*) is the posterior process. The free energy is a function of the parameters and a functional of the approximating distribution *q *(**x**). Since the posterior process is also Markovian, an optimisation where *q *is unconstrained would return the true posterior. Unfortunately, it would also require the solution of the Master equation (and of the corresponding backward equation) which, as we remarked before, is not computationally feasible.

The key idea of the variational approximation is to restrict the class of approximating distributions *q*. In particular, we will make a mean field approximation so that the approximating process is factorised across different species. This means that we will impose(7)

where  are the rates for the approximating process. If *f *(**x**'|**x**) are the rates for the prior process, it was shown in [10] that the free energy (6) for this choice of approximating process is given by(8)

Here(9)

are the mean-field rates obtained by averaging the rates of the prior process under the approximate posterior for all species but the one under consideration. Opper and Sanguinetti [10] then went on to derive an iterative functional gradient descent algorithm to minimise the free energy w.r.t. the approximating distribution *q*. Parameter estimation was then performed in an M-step returning maximum likelihood point estimates.

Our model has a relatively low number of parameters due to the fact that the decay and diffusion parameters are shared across all species. This means that we can efficiently use a sampling approach to obtain an estimate of the full posterior distribution over the parameters. Specifically, we set a prior *p *(***θ***) over the parameters and sample from the distribution(10)

where the variational free energy is used as an approximation to the (intractable) true joint over the data and parameters. This allows us to use a Metropolis-Hastings sampler to obtain an approximation to the posterior distribution over the parameters.

## Authors' contributions

VK, MO and GS designed the research. MD and GS implemented the research and analysed the results. All authors wrote the paper. All authors read and approved the final manuscript
